# Obesity and gynecological cancers: A toxic relationship

**DOI:** 10.1002/ijgo.13870

**Published:** 2021-10-20

**Authors:** Ignacio A. Wichmann, Mauricio A. Cuello

**Affiliations:** ^1^ Division of Gynecology and Obstetrics School of Medicine Pontificia Universidad Católica de Chile Santiago Chile; ^2^ Department of Obstetrics School of Medicine Pontificia Universidad Católica de Chile Santiago Chile; ^3^ Advanced Center for Chronic Diseases Pontificia Universidad Católica de Chile Santiago Chile; ^4^ Department of Gynecology School of Medicine Pontificia Universidad Católica de Chile Santiago Chile

**Keywords:** carcinogenesis, endometrial neoplasms, FIGO Cancer Report, obesity, ovarian neoplasms, prognoses, treatment outcome, uterine cervix neoplasms

## Abstract

Despite the evidence supporting the relevance of obesity and obesity‐associated disorders in the development, management, and prognosis of various cancers, obesity rates continue to increase worldwide. Growing evidence supports the involvement of obesity in the development of gynecologic malignancies. This article explores the molecular basis governing the alteration of hallmarks of cancer in the development of obesity‐related gynecologic malignancies encompassing cervical, endometrial, and ovarian cancers. We highlight specific examples of how development, management, and prognosis are affected for each cancer, incorporate current knowledge on complementary approaches including lifestyle interventions to improve patient outcomes, and highlight how new technologies are helping us better understand the biology underlying this neglected pandemic.

## Synopsis

This review focuses on how obesity impacts cancer hallmarks in gynecologic malignancies, thus affecting the diagnosis, management, treatment, and prognosis of these diseases.

## 1 INTRODUCTION

**FIGURE 1:**
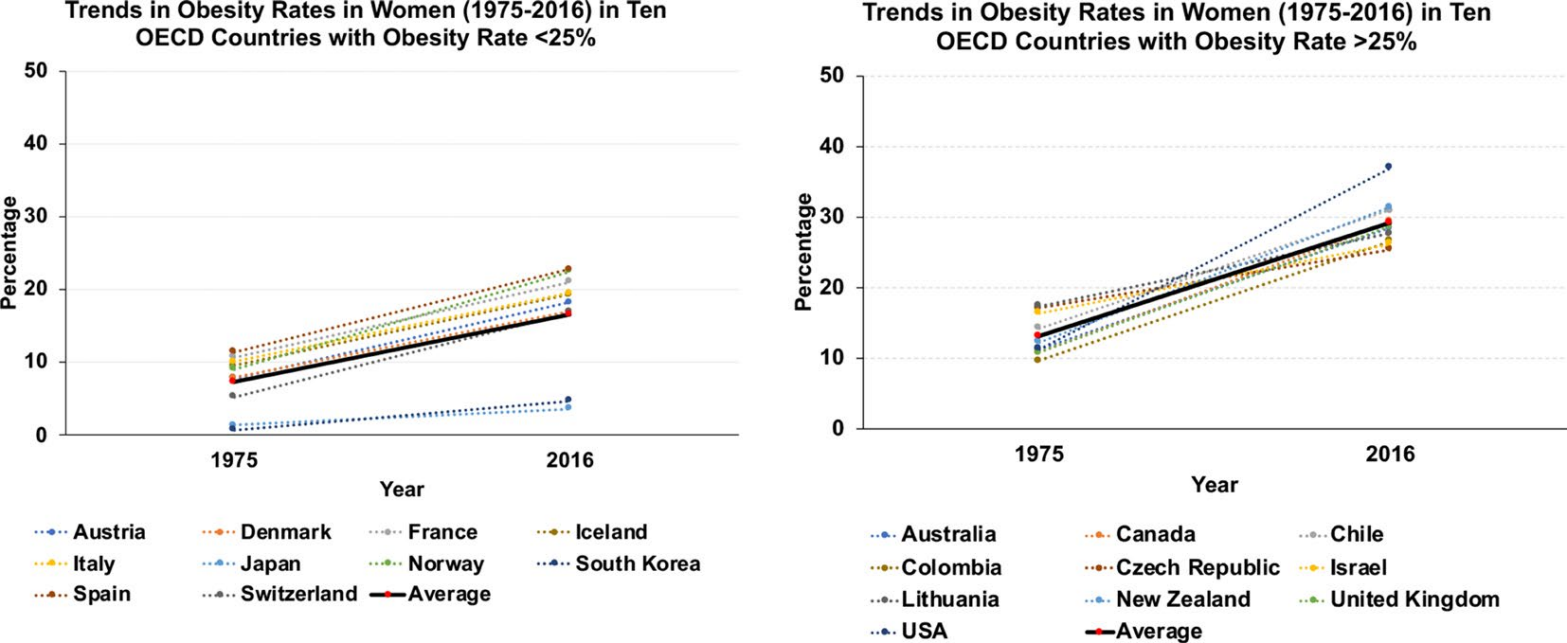
Trends in obesity rates among women from 20 OECD countries between 1975 and 2016. On the left, trends in countries that currently present obesity prevalence less than 25%. On the right, countries where current prevalence exceeds 25%. The continuous black line (red dots) indicates the average rate trend for 10 countries analyzed. Graphs were built after downloading data from OurWorldInData.org ^7^.

Since 2020, the world has been hit by the coronavirus (COVID‐19) pandemic, a pandemic that has triggered the global community into implementing massive efforts to reduce transmission of the disease.^1^, ^2^, ^3^ One of the major risk factors determining worse prognosis in COVID‐19 is obesity.^4^, ^5^ Obesity is a latent disease that was declared by the World Health Organization (WHO) in 2015 as a noninfectious and noncommunicable pandemic, and its prevalence trends and disease‐related morbidity and death have continued to rise in both sexes worldwide. By 2012, 37% of reproductive‐age (25–54 years) women in the USA were obese.^6^ In fact, prevalence of obesity in women tripled in low‐, middle‐, and high‐income countries from 1975 to 2016 (Figure 1).

The Global Burden of Disease study showed that 4.7 million people died prematurely in 2017 because of obesity‐related diseases.^8^ Put into context, WHO global estimates showed that around 650 million people were obese in 2016 (15% of women aged 18 years or over) and that 8% of total deaths in one year were obesity related. This number represents roughly 50% more events than the total amount of COVID‐19 deaths in 1.5 years of the pandemic.

Despite these numbers, the relevance of obesity seems to be neglected. Together with hyperglycemia, hypertension, and low levels of high‐density lipoprotein (HDL) cholesterol, these alterations make up the main components of metabolic syndrome, a condition that has been associated with increased morbimortality including type 2 diabetes and cancer.^9^, ^10^, ^11^ This is particularly worrisome, as obesity and hyperglycemia play a pivotal role in tumorigenesis^10^, ^12^ and maternal obesity has been associated with all cancer outcomes in human offspring.^6^ In addition, previous reports have shown that higher body mass index (BMI) values correlate with significant increasing trends for death from breast, uterus, cervical, and ovarian cancers in women.^13^

**FIGURE 2:**
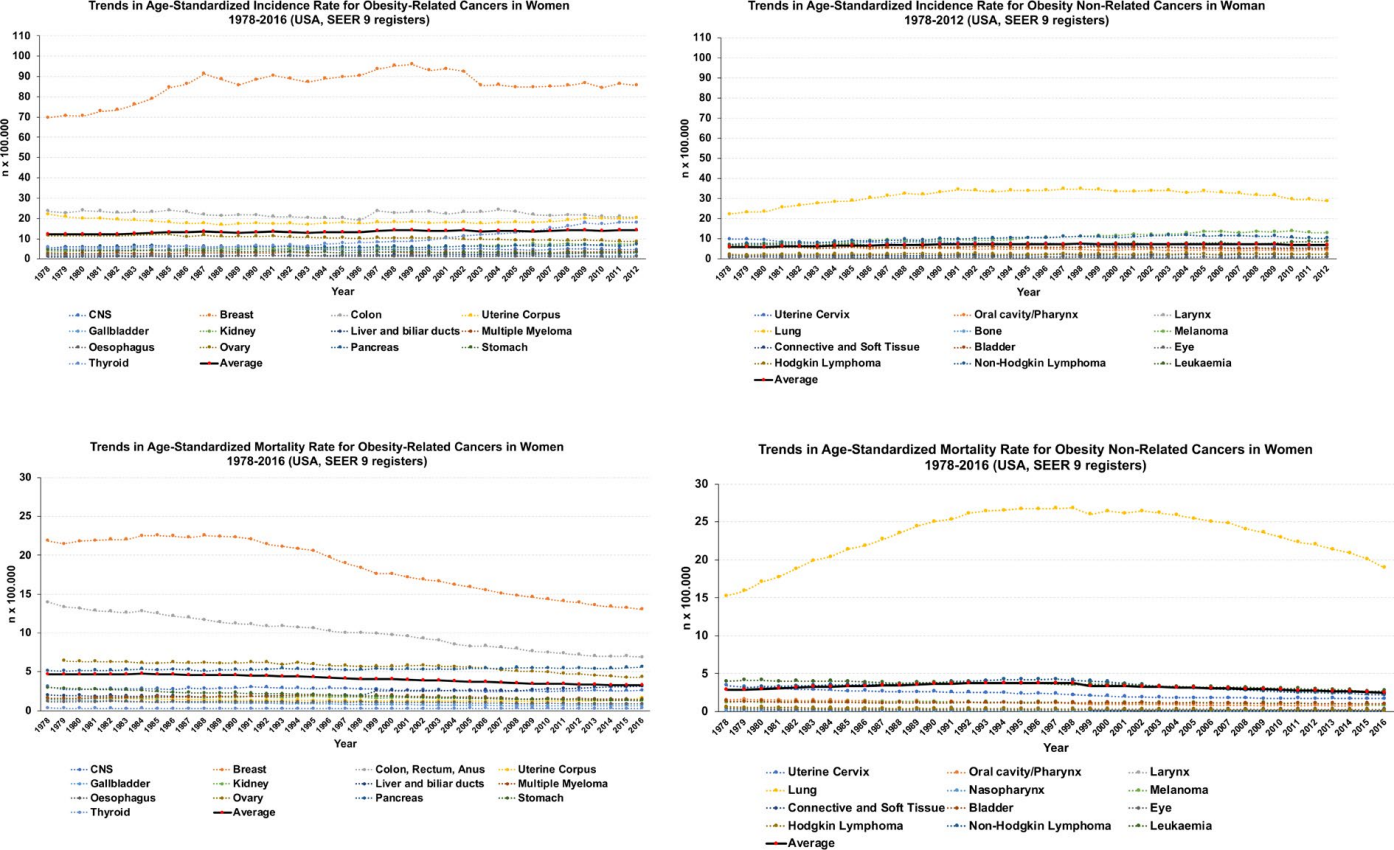
Trends in age‐standardized incidence and mortality rates for obesity‐related and nonrelated cancers among women between 1978 and 2016 in the USA (according to nine SEER registers). Top graphs summarize trends in incidence rates. Bottom graphs show trends in mortality rates. The continuous black line (red dots) indicates the average rate trend for all cancers analyzed. Graphs were built after downloading data from the Global Cancer Observatory ^17^, ^18^, ^19^.

In recent years, important scientific advances have allowed us to improve our understanding of the molecular bases governing many cancers, including those of gynecologic origins.^14^, ^15^ Similar advances made in preventive and diagnostic medicine, surgery, radiotherapy, and chemotherapy have allowed us to tailor and test targeted therapies (i.e. poly ADP‐ribose polymerase [PARP] inhibitors), immunotherapy, and to evolve towards precision medicine approaches.^14^, ^15^, ^16^ Regardless of all these improvements, incidence and mortality rates of obesity‐related cancers have not improved significantly in the last 30 years. As shown in Figure 2, the average incidence rate for obesity‐related cancers is higher and shows an increasing trend among American women.

**FIGURE 3:**
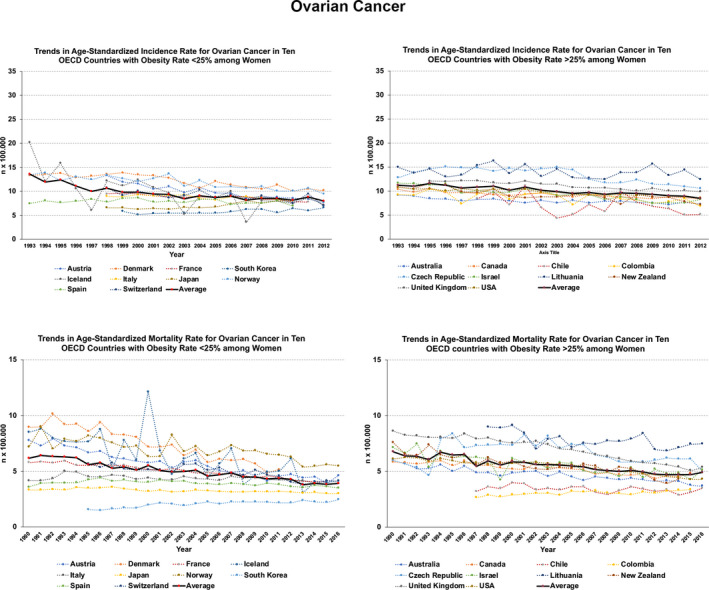
Trends in age‐standardized incidence (1993–2012) and mortality (1990–2016) rates for ovarian cancer in 20 OECD countries. On the left, trends in countries that currently present obesity prevalence less than 25%. On the right, those countries where current prevalence exceeds 25%. The continuous black line (red dots) indicates the average rate trend for 10 countries analyzed. Graphs were built after downloading data from the Global Cancer Observatory ^17^, ^18^, ^19^.

**FIGURE 4:**
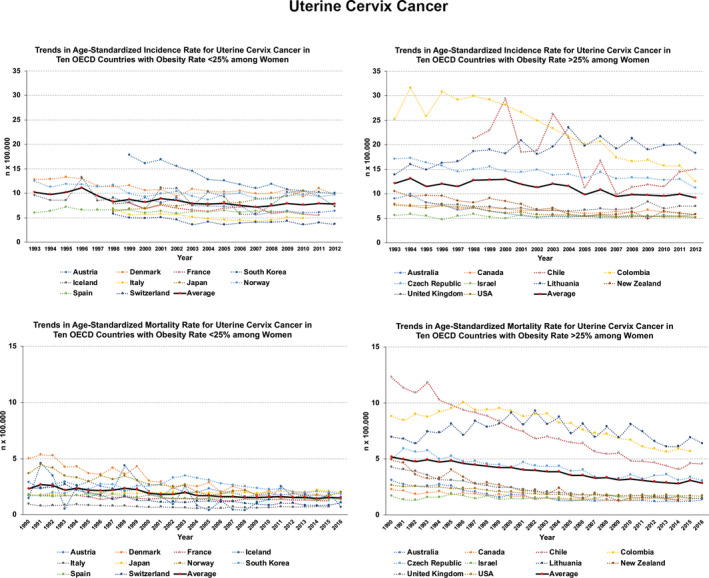
Trends in age‐standardized incidence (1993–2012) and mortality (1990–2016) rates for uterine cervix cancer in 20 OECD countries. On the left, trends in countries that currently present obesity prevalence less than 25%. On the right, those countries where current prevalence exceeds 25%. The continuous black line (red dots) indicates the average rate trend for 10 countries analyzed. Graphs were built after downloading data from the Global Cancer Observatory ^17^, ^18^, ^19^.

**FIGURE 5:**
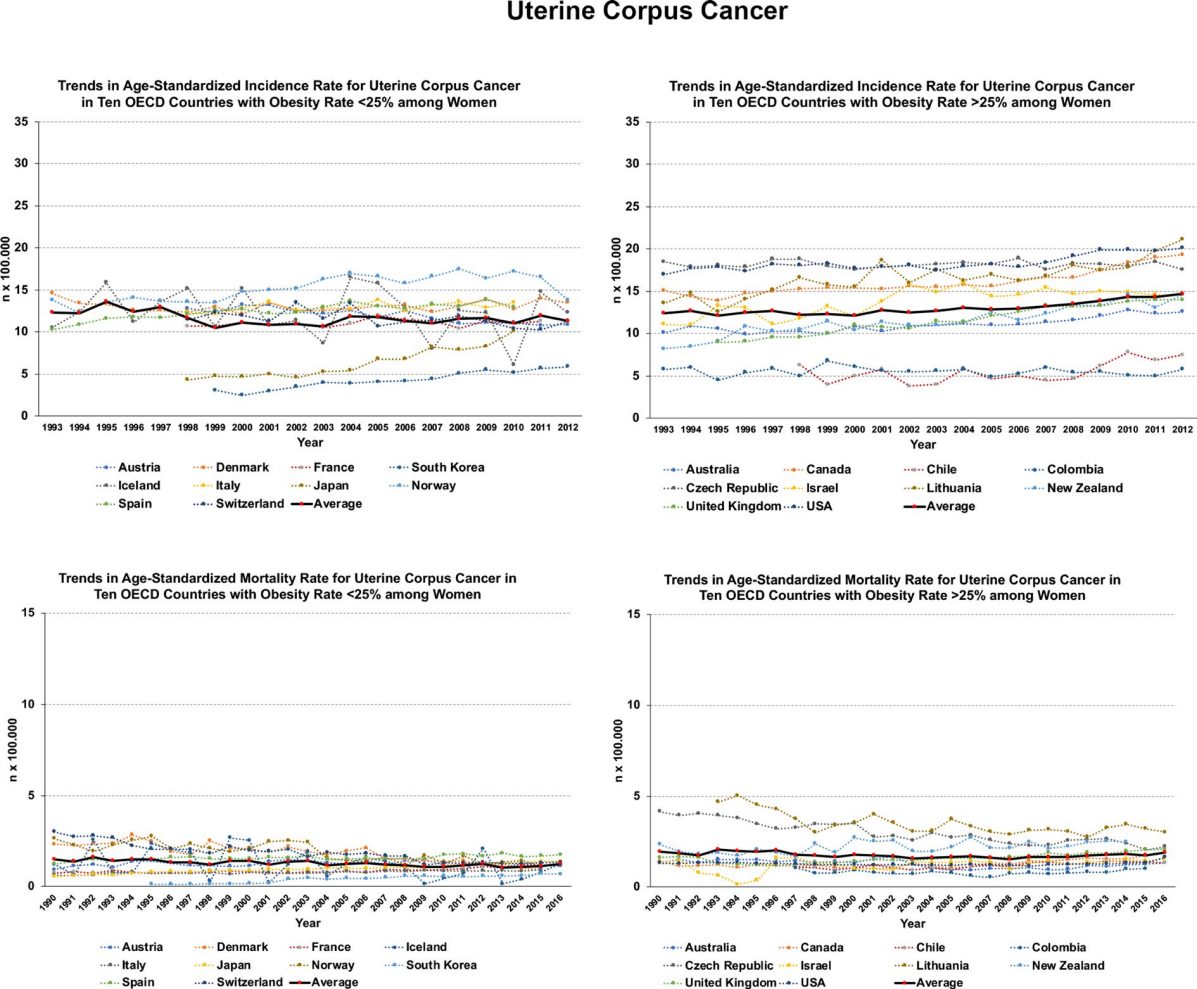
Trends in age‐standardized incidence (1993–2012) and mortality (1990–2016) rates for uterine cancer (endometrial) in 20 OECD countries. On the left, trends in countries that currently present obesity prevalence less than 25%. On the right, those countries where current prevalence exceeds 25%. The continuous black line (red dots) indicates the average rate trend for 10 countries analyzed. Graphs were built after downloading data from Global Cancer Observatory ^17^, ^18^, ^19^.

Specific analysis of incidence and mortality rates and trends in 20 OECD countries (10 with current obesity prevalence under 25% and 10 over 25%) spotlights some relevant issues in gynecologic cancers. For ovarian cancer, both rates remain relatively stable or increase. This is evident for the mortality rates among countries with higher prevalence of obesity (Figure 3). For uterine cervix cancer, rates and trends are worse among countries with higher obesity rates (Figure 4). For uterine corpus cancer, rates and trends are even worse for countries with higher obesity rates and there exists an evident increasing trend in incidence and mortality (Figure 5).

**FIGURE 6:**
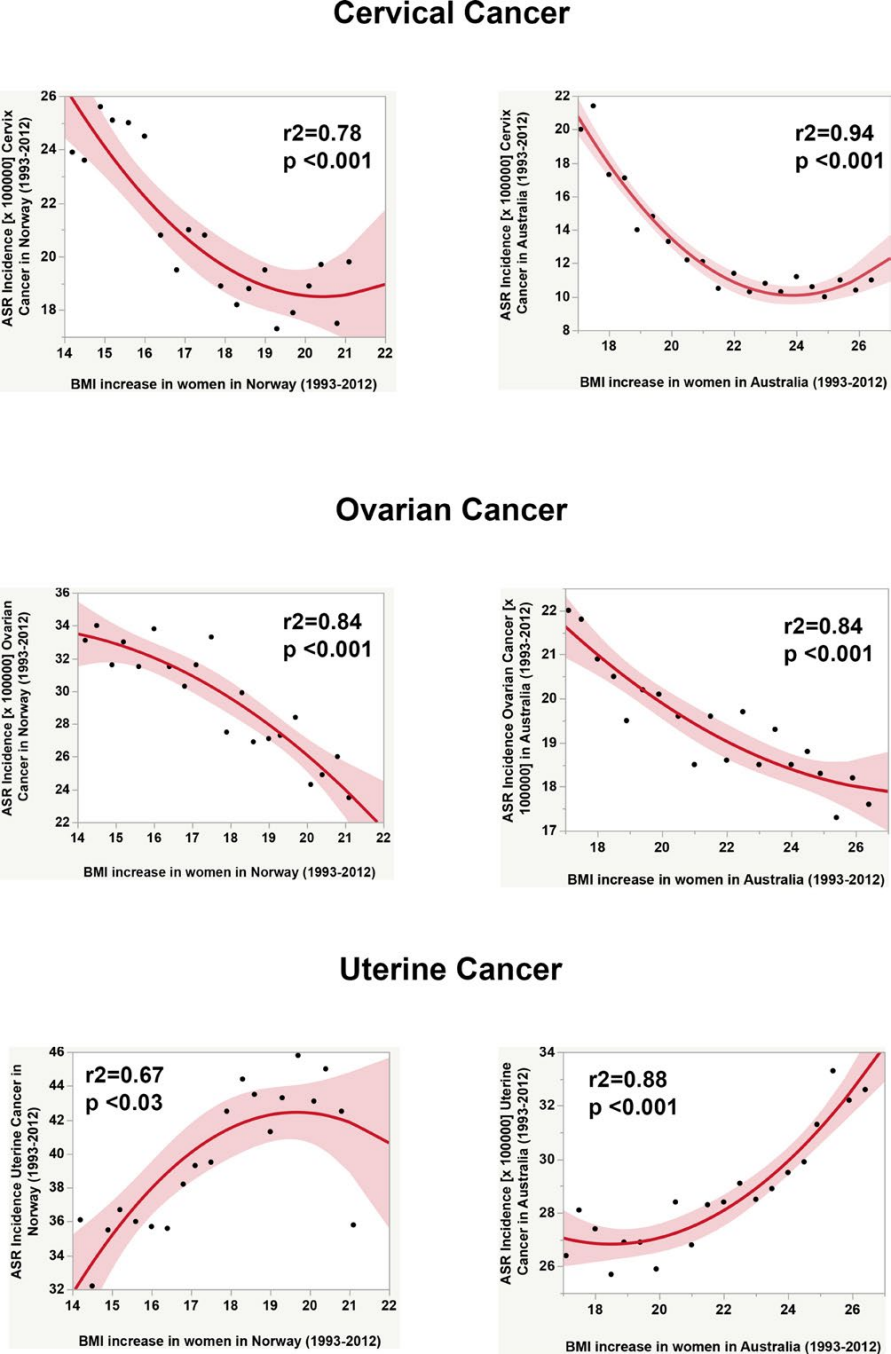
Comparative analysis of the effect of increase in body mass index among women (35– to +85‐year‐olds) in age‐standardized incidence rates for three gynecologic cancers over time (1993–2012) between Norway (country with obesity prevalence less than 25% in 2016) and Australia (country with obesity prevalence over 25% in 2016). The continuous red line and surrounding shadows show the fitted polynomial (quadratic) line and its 95% CI shaded fit, respectively. Graphs were built after downloading data from OurWorldInData.org ^7^ and the Global Cancer Observatory ^17^, ^18^, ^19^. Correlation analyses were made using JMP16 software (SAS Institute, Cary, NC, USA).

**FIGURE 7:**
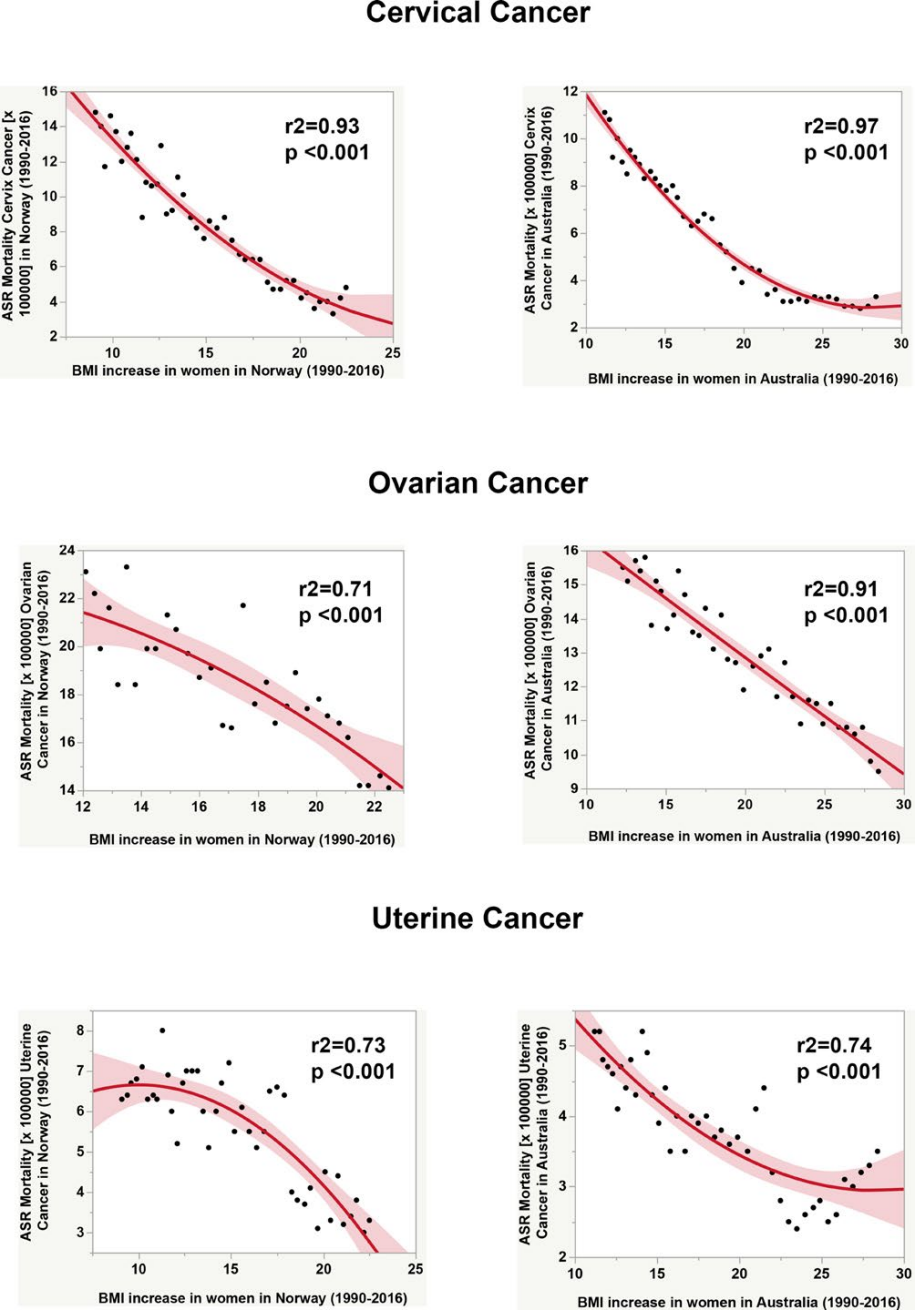
Comparative analysis of the effect of increase in body mass index among women (35– to +85‐year‐olds) in age‐standardized mortality rates for three gynecologic cancers over time (1993–2012) between Norway (country with obesity prevalence less than 25% in 2016) and Australia (country with obesity prevalence over 25% in 2016). The continuous red line and surrounding shadows show the fitted polynomial (quadratic) line and its 95% CI shaded fit, respectively. Graphs were built after downloading data from OurWorldInData.org ^7^ and the Global Cancer Observatory ^17^, ^18^, ^19^. Correlation analysis was made using JMP16 software (SAS Institute, Cary, NC, USA).

To address how the progressive increase in BMI impacts on the incidence and mortality rates of gynecologic cancers, we carried out regression analyses using data from two countries with good long‐term registers and different obesity prevalence rates: Australia (over 25%) and Norway (less than 25%). Figures 6 and 7 depict the dramatic effects of obesity for uterine corpus cancer, with increasing incidence rates over time as obesity continues to rise.

These reports spotlight the association between obesity and increased cancer risk in gynecologic cancers.^6^ The main goal of this review is to summarize the main molecular mechanisms through which obesity contributes to development and affects therapeutic response and patient outcomes in gynecologic cancers. In addition, we identify and expose unanswered questions that warrant further research to modify the current scenario.

## 2 OBESITY AND HALLMARKS OF CANCER

**FIGURE 8:**
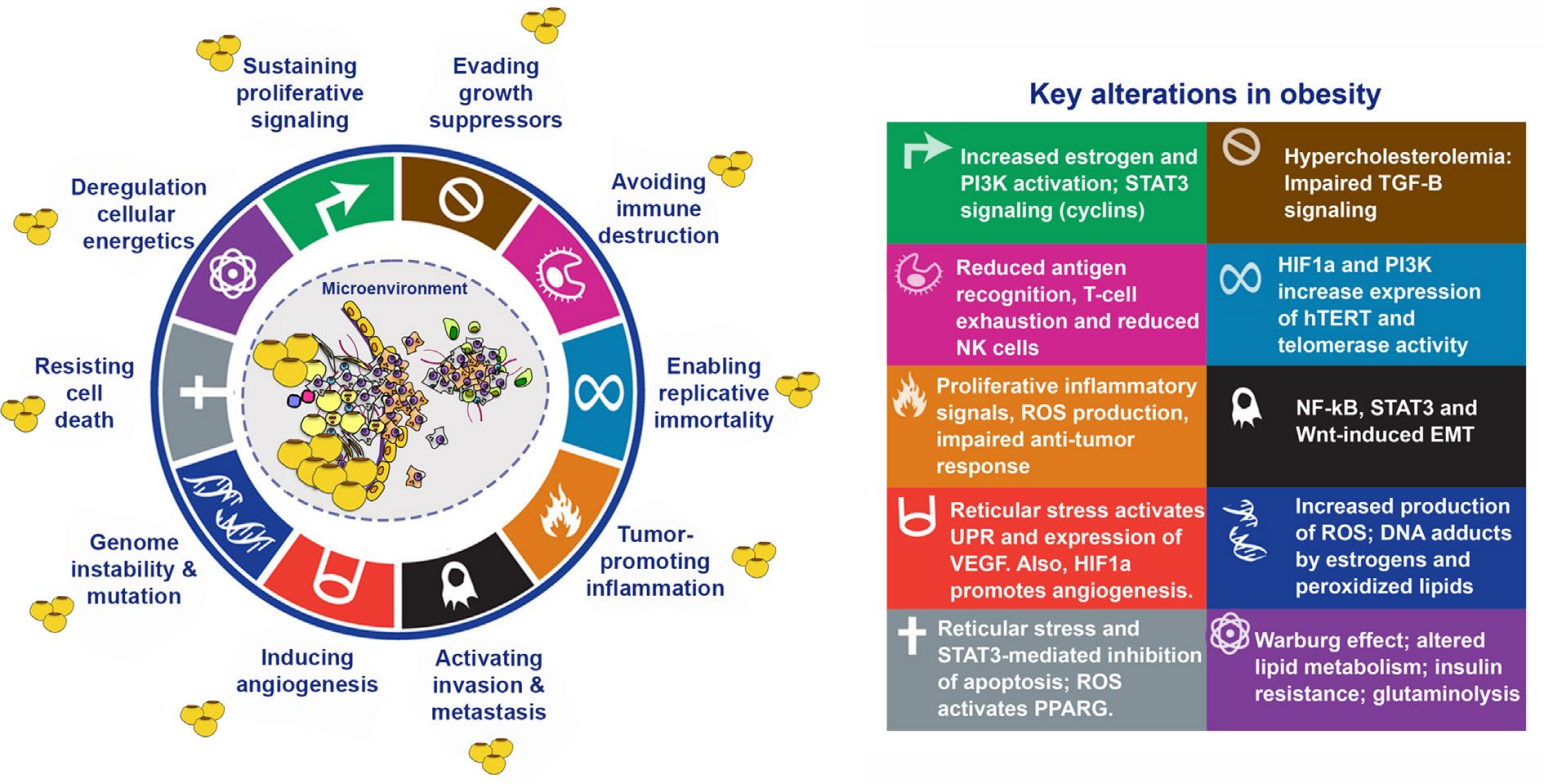
Effects of obesity and adiposity in the hallmarks of cancer. The three stuck and sketched yellow and brown circles symbolize hypertrophic and dysfunctional adipocytes. Modified from Hannah and Weinberg ^14^, © 2011, with permission from Elsevier.

Obesity impacts cancer hallmarks through different mechanisms (Figure 8). In gynecological cancers, this occurs mainly through alterations in hormonal, inflammatory, and metabolic pathways.^20^ Increased estrogen signaling, obesity‐related insulin resistance, and chronic low‐grade inflammation contribute in concert to stimulate anabolic processes, inhibit apoptosis, and stimulate cell proliferation, in part, by altering the mitogenic PI3K/AKT/mTOR pathway. Additionally, obesity leads to major alterations in the lipidic composition of organelles and membrane dynamics which may further contribute to the alteration of cancer hallmarks.^21^ Some of the key mechanisms orchestrating alterations in cancer hallmarks are summarized in this section.

1. **Sustaining proliferative signaling**. One of the principal traits of cancer cells is sustaining proliferative signalling.^14^ Adipocyte hypertrophy determines increased expression of aromatase which converts androgens to estrone and estradiol.^20^ Additionally, sex hormone‐binding globulin levels decrease in obesity, resulting in increased availability of bioactive estrogens. These conditions favor signal transduction by the estrogen α and β receptors leading to activation of the estrogen signaling cascade and downstream activation of the mitogenic PI3K/AKT/mTOR signaling pathway.^20^ A central pathway for cell proliferation, this pathway is frequently hyperactivated in many cancers and obesity. Estrogen, insulin, and proliferative inflammatory signaling activate this pathway in obesity.^22^ Additionally, chronic low‐grade inflammation results in activation of intracellular signaling pathways encompassing nuclear factor‐ĸB (NF‐ĸB), which regulates interleukin 6 (IL‐6). In turn IL‐6, via its receptor and intracellular cascade mediated by Janus kinase (JAK) proteins, activates signal transducer and activator of transcription 3 (STAT3), which results in expression of genes that include cyclins, thus inducing cell proliferation.^23^ Obesity is also associated with reticular stress and remodeling, and its lipidic composition is altered in obesity. These conditions play a relevant role in proliferative signaling in obesity.^20^
  In aggregate, these observations spotlight the functional interplay linking obesity, insulin signaling, increased estrogens, and chronic inflammation with sustaining proliferative signaling in gynecological cancers.^20^

- **Evading growth suppression**. Raft‐mediated transforming growth factor‐β (TGF‐β) signaling negatively regulates cell proliferation. TGF‐β signaling is impaired by raft composition alterations, leading to rapid differentiation and cell proliferation. Interestingly, cholesterol can decrease binding of TGF‐β to its receptor and impair signaling by regulating endocytosis and degradation.^21^ Therefore, intervention of cholesterol homeostasis by cholesterol‐lowering drugs such as statins can be of particular interest in patient management in obesity‐associated cancers.
- **Avoiding immune destruction**. The capacity of the tumor cell to evade the immune antitumor response mechanisms is regarded as one of the key features and hallmarks of cancer cells.^14^ Obesity reduces the diversity of T cell receptors (TCRs) on circulating T cells, impacting the number of recognizable antigens. Lymph node size is reduced, as well as the migration of dendritic cells to the lymph nodes and the number of T cells in the lymph nodes. One additional mechanism that may be implicated in the impaired maturation of T cells and their reduced capacity to recognize antigens in obesity is altered composition of membrane rafts. Raft‐dependent pathway maturation of T cells is altered in obese patients.^21^ Furthermore, suppression of anti‐inflammatory pathways enables activation of dendric cells within the white adipose tissue and persistent antigen presentation by dendric cells to T cells. This may lead to T cell exhaustion and reduced T cell effector response.^24^ Accordingly, a recent study by Porsche et al.^25^ has shown that obesity causes T cell exhaustion within the adipose tissue that is dependent on localized soluble factors and cell‐to‐cell interactions. Hypercholesterolemia in obese patients hinders the differentiation of hematopoietic stem cells, resulting in negative regulation of natural killer cell production. Additionally, lipid storages in the form of lipid droplets in dendritic cells impair antigen presentation and immunity. This is because lipid droplets act as major eicosanoid reservoirs in the cell, leading to alterations of the immune response. In fact, lipid droplet content alters antigen presentation in a cell type‐specific fashion, as well as chemotaxis and phagocytosis.^21^
- **Enabling replicative immortality**. Increased telomerase activity in cancer cells grants them the ability of unlimited replication.^14^ Activated hypoxia‐inducible factor 1α (HIF‐1α) and phosphoinositide 3‐kinase (PI3K) signaling contribute to upregulation of human telomerase reverse transcriptase (hTERT) in cancer cells.^21^ These pathways are upregulated in obesity.^26^, ^27^
- **Tumor‐promoting inflammation**. Together with genome instability and mutation, tumor‐promoting inflammation is one of the most relevant enabling characteristics of cancer cells, as it modulates an important fraction of the remaining hallmarks.^14^
  The adipose tissue harbors a rich immune cell population that is heavily affected by obesity. Hypertrophic adipocytes secrete increased amounts of monocyte chemoattractant protein‐1 (MCP‐1) and tumor necrosis factor α (TNF‐α), resulting in enhanced accrual of macrophages to the adipose tissue. In addition, the hypertrophic adipocytes from obese individuals suffer from mitochondrial and reticular stress, subsequently leading to upregulation of proapoptotic Fas and its ligand Fas‐L, and cell death. Apoptotic adipocytes release damage‐associated molecular patterns (DAMPs) that activate resident macrophages which surround the adipocytes, forming crown‐like structures (CLS). CLS favor polarization of macrophages towards a proinflammatory M1 phenotype.^24^ These activated inflammatory cells secrete and increase production of reactive oxygen species, predisposing inflamed tissues to DNA breaks and mutations.
  Additionally, hypertrophic adipocytes and activated macrophages secrete a variety of macrophage‐recruiting and proinflammatory factors including leptin, IL‐6, TNF‐α, interferon γ (IFNγ), and MCP‐1, thus perpetuating proinflammatory conditions in the adipose tissue. IL‐6 production is mainly regulated by NF‐ĸB, which also regulates signaling pathways involved in inflammation, proliferation, and expression of pro‐survival genes. IL‐6 has a well‐established role in the activation of immune cells. Aside from immune cell activation, interaction of IL‐6 with the IL‐6R results in the activation of glycoprotein 130 (gp130) and downstream activation of JAK proteins and signal transducer and activator of STAT3. The IL‐6/JAK/STAT3 pathway has marked effects on the antitumor immune response, exerting an inhibitory effect on neutrophils, natural killer cells, effector T lymphocytes, and antigen‐presenting dendritic cells. Therefore, hyperactivation of this pathway seems to have a relevant role in the reduction of the antitumor immune response.^23^
  Aside from these mechanisms, lipid droplets also affect major inflammatory pathways. Lipid droplets are a major site of eicosanoid synthesis, from which a plethora of cytokine amplifiers originate. The composition of anti‐ or proinflammatory molecules determines the type of inflammatory response. Therefore, the altered composition of these molecules in obesity triggers a proinflammatory response that favors tumor formation.^21^
  Finally, inflammation can impair insulin signaling generating insulin resistance. Increased insulin secretion promotes cell proliferation via stimulation of the PI3K/AKT/mTOR pathway.^22^
- **Activating invasion and metastasis**. NF‐ĸB and STAT3 have been implicated in epithelial‐to‐mesenchymal transition (EMT), the process by which cancer cells become more invasive and acquire metastatic potential and genomic instability due to impaired DNA repair mechanisms and increased DNA damage.^28^ The Wnt signaling pathway is also of pivotal importance in cancer development, as it is both relevant in the acquisition of cancer stem‐like cell traits and induction of EMT. Post translational lipid modifications of the Wnt ligands are essential for correct interaction with its frizzled (Fzd) receptors and activation of the pathway. Specifically, Wnt11 interaction with Fzd8 has been described to induce TGF‐β induced EMT.^21^
- **Inducing angiogenesis**. Altered endoplasmic reticulum membrane composition and reticular stress in obesity activate the unfolded protein response (UPR) that binds directly to the vascular endothelial growth factor (VEGF) promoter and induction of angiogenesis by the tumor‐associated adipocyte.^13^ Further, VEGF stimulates the UPR in a positive feedback loop.^21^ Additionally, hypertrophic adipose tissue contribute to the reduction of available oxygen and induction of HIF‐1α, promotion of angiogenesis, and tumor dissemination.^10^
- **Genome instability and mutation**. This hallmark, together with tumor‐promoting inflammation, is considered one of the enabling hallmarks of cancer, regulating other hallmarks.^6^ Mutations induced by inflammatory cell‐derived reactive oxygen species are known mutagens affecting genomic stability of cancer cells. These reactive oxygen species also induce lipid peroxidation and generation of reactive lipid peroxide species, which can form DNA adducts and induce double‐strand breaks.^21^ Increased estrogen levels in obesity also contribute to genome instability. Estrogen metabolites are also known to interact with DNA and generate adducts, leading to cumulative DNA damage and genetic instability through double‐strand DNA breaks.^20^ Thus, lipids and estrogens play an additional role in cancer initiation by acting as mutagens.
  Moreover, endometrial, cervical, and ovarian cancers feature some of highest mutational frequencies in the PI3K/AKT/mTOR mitogenic pathway,^22^ highlighting its relevance in gynecologic malignancies.^21^, ^28^
- **Resisting cell death**. The adipose tissue of some obese cancer patients displays altered long fatty acid levels associated with peroxisome stress and oxidation—a condition that leads to accumulation of reactive oxygen species and activation of the peroxisome proliferator‐activated receptor (PPAR) pathway. Downstream effects include inhibition of apoptosis and increased cell proliferation.^17^ Additionally, reticular stress‐dependent activation of the UPR transcription factors in obesity also leads to alteration of antiapoptotic proteins XBP1, ATF6f, and ATF4.^17^, ^25^
  STAT3 increases survival via upregulating antiapoptotic proteins.^28^
- **Deregulating cellular energetics**. Cancer cells are known to acquire altered metabolic states favoring aerobic glycolysis over oxidative phosphorylation, a phenomenon termed the Warburg effect. Together with increased glutaminolysis and altered lipid metabolism, they make up the key components in cancer metabolic reprogramming, one of the hallmarks of cancer.^10^, ^14^ Metabolic reprogramming is a complex phenomenon orchestrated by several alterations including activation of KRAS, mTOR, MYC, p53. Mutations in mitochondrial DNA and hypoxic conditions that activate HIF‐1α can cause mitochondrial dysfunction or inhibition of the mitochondrial respiratory chain, also favoring aerobic glycolysis.^20^ Another relevant aspect of this hallmark in obesity is the association it has with insulin resistance. Given that insulin regulates clearance of glucose from the blood, insulin resistance determines postprandial hyperglycemia and hyperinsulinemia.^22^ Aside from its effects on glucose homeostasis, excess insulin activates the PI3K pathway, contributing to progression of the disease via increased cell proliferation and inhibition of apoptosis.^30^

## 3 OBESITY AND DEVELOPMENT OF GYNECOLOGIC CANCERS

So far, there is no doubt on the independent and positive correlation between increase in BMI and the risk of developing endometrial adenocarcinoma, particularly the type 1 or endometrioid variant.^31^ These tumors are estrogen respondent and usually develop within a hyperplastic epithelium. Conversely, type 2 tumors are less responsive to estrogens and develop within an atrophic background. Obesity increases the risk of type 1 tumors by roughly three‐fold and almost two‐fold for type 2 tumors. Metabolic syndrome also doubles the risk of developing endometrial cancer in both pre‐ and postmenopausal women, most likely due to estrogen independent activation of the PI3K pathway. Notably, obesity associations have not been clearly proven for cervical, ovarian, vaginal, or vulvar cancers.^32^, ^33^ Possibly, the inclusion of some histologies in which obesity did not exert a role during earlier stages of carcinogenesis may mask this relationship. More recent studies analyzing specific histologies in these cancers have begun to establish this relationship with obesity. In cervical cancer, an increase in BMI has been associated with a higher risk of developing cervical adenocarcinoma.^34^, ^35^ Additionally, persistent cervical infection by high‐risk HPV strains is favored in obese women presenting vaginal dysbiosis, which is characterized by an increase in microbial diversity that prompts malignant transformation of the cervical epithelium.^36^, ^37^, ^38^, ^39^ For epithelial ovarian cancer, a disease including at least five histological subtypes and arising from fallopian tube fimbria, ovarian, and peritoneal surfaces, most recent studies have proven a relationship between increasing BMI and nonserous histologies.^40^, ^41^ For serous histologies, obesity would increase the risk of those arising from the peritoneum.^42^ Summarizing the effects of weight gain on the development of cancer, a recent meta‐analysis addressed the impact of 5 kg weight gain on the relative cancer risk in adults.^43^ Results showed that the overall relative risk of 5 kg weight gain was 1.11. Specifically for gynecologic cancers in postmenopausal women, the relative risks were 1.39 (95% CI, 1.29–1.49) for endometrial cancer in nonusers of hormone replacement therapy (HRT), 1.09 (95% CI, 1.02–1.16) for endometrial cancer in HRT users, and 1.13 (95% CI, 1.03–1.23) for ovarian cancer patient nonusers of HRT. These findings show the magnitude of the association between weight gain in postmenopausal women and development of gynecological cancers. For other gynecologic cancers such as vulvar and vaginal cancer, higher risk has been associated with metabolic syndrome.^44^

## 4 EFFECTS OF OBESITY ON THE DIAGNOSIS OF GYNECOLOGIC CANCERS

Obesity is related to diagnosis at earlier ages only in endometrial cancer.^45^ Obesity seems to affect clinical presentation of symptoms, contributing to a delayed diagnosis in ovarian cancer.^46^ Roughly three‐quarters of all ovarian cancer patients are diagnosed at late stages of the disease, with a 5‐year survival rate of 50%.^20^ For cervical cancer, obese and morbidly obese status entail a higher rate of defective screening, inadequate clinical assessment, and higher risk of missing hidden or partially visible lesions.^47^ In fact, obese patients are often diagnosed at advanced stages of the disease despite regular examination and are twice more likely to develop cervical cancer than lean patients.^20^ Beyond tumor biology, obesity constitutes a morbid condition with sociocultural implications which is more prevalent among underprivileged (low income and less educated) communities. Lack of knowledge and limitations in healthcare access undoubtably contribute to delayed diagnosis and subsequent management of gynecologic cancers.

## 5 MANAGEMENT OF GYNECOLOGIC CANCERS AMONG OBESE AND MORBIDLY OBESE WOMEN

Obesity does not commonly make it difficult to obtain an adequate biopsy or perform image staging (i.e. MRI, CT, or PET/CT scan) of gynecologic cancers. Once confirmed and if the extension of the disease allows it, the first option will be the complete surgical removal of the tumor coupled with adequate staging. However, in some cases, the presence of obesity may cause the medical team to opt for nonsurgical management or may determine suboptimal surgery or a more complex perioperative management scenario (i.e. high‐cost instrumentation/appliances; use of robotic rather than laparoscopic surgery or laparotomy; higher rates of intraoperative, immediate postoperative and 30‐day complications; and hospital readmissions), delaying adjuvant therapies, among other possibilities affecting the prognosis. In this respect, Inci et al.^48^ have recently identified overweight and obesity as significant predictors of postoperative complications. Similarly, Pyrzak et al.^49^ identified a BMI of 30 or higher as a risk factor for complication‐related 30‐day hospital readmission. It is well known that obesity entails a higher risk of thromboembolism and wound infection, particularly for open, staging, or cytoreductive and time‐extended surgeries.^50^, ^51^, ^52^, ^53^

With respect to high‐grade serous ovarian cancer (HGSOC), achieving optimal debulking, primarily or after neoadjuvant chemotherapy, constitutes an independent and relevant prognosis factor.^54^ Recently, Wang et al.^55^ have identified five molecular subtypes of HGSOC. One of them, the mesenchymal subtype, is the less optimally debulked and exhibits the poorest outcome. Our group has recently found that high leptin levels as seen among obese women induce EMT in HGSOC cell lines.^56^ In addition, we have demonstrated that HGSOCs overexpressing obesity and lipid metabolism‐related genes share significant lower chances of achieving optimal debulking and having positive outcomes.^57^ This group is enriched for the mesenchymal subtype.^58^

Radiotherapy either alone or in combination with chemotherapy constitutes the primary treatment for those gynecologic cancers not suitable for surgery or those locally advanced (i.e. uterine cervix cancer). It is also indicated as adjuvant therapy in cases harboring risk factors for local recurrence and as part of palliative care. Obesity, particularly extremely morbid obesity, poses a major hindrance to treatment planning, limits the use of common radiotherapy equipment (commonly designed to support certain BMI range and physical characteristics of individuals), and increases the risk of gynecologic and cutaneous radiation‐related toxicities.^59^, ^60^

Chemotherapy administration and efficacy are also challenged by obesity. Obese cancer patients receiving chemotherapy have worse clinical outcomes. Potential explanations for these adverse results include differences in pharmacokinetics, metabolic dysregulation, induction of chemoresistance, or clinicians’ decisions to reduce dose intensity during treatment to minimize toxicities.^61^, ^62^ Since 2012, American Society of Clinical Oncology (ASCO) guidelines recommend using actual body weight for dosing in all patients treated with curative intent, irrespective of obesity, to avoid compromising clinical outcomes. Outcomes in obese patients are no different to lean patients when the correct dose is administered.^63^

## 6 CLINICAL OUTCOMES AMONG OBESE GYNECOLOGICAL CANCER PATIENTS

Obesity appears to have a negative impact for all gynecologic cancers regarding prognosis and treatment outcomes.^32^ In endometrial cancer, Donkers et al.^64^ demonstrated that obesity and higher visceral fat percentage were associated with poor overall and disease‐specific survival (*p* = 0.006 and *p* = 0.026, respectively) in nonendometrioid histologies but not in high‐grade endometrioid tumors. Similarly, Mauland et al.^65^ demonstrated that tumors arising in lean patients had better outcomes and showed enrichment of gene sets related to immune activation and inflammation. Therefore, the global metabolic setting seems to be determinant of the antitumor immune response. Recently, a multivariate analysis of over 1000 cervical cancers by Gnade et al.^47^ demonstrated that higher BMI was associated with worse overall survival (*p* < 0.01) in both obese (HR 1.25; 95% CI, 0.92–1.69) and morbidly obese women (HR 2.27; 95% CI, 1.56–3.31). Another study by Clark et al.^66^ demonstrated worse overall survival for obese and overweight than normoweight cervical cancer patients (22.2 vs 28.4 months, *p* = 0.03) and a trend toward worse disease‐specific survival (21.9 vs 28.4 months, *p* = 0.09) in a cohort of 632 cases. In epithelial ovarian cancer, overweight and obesity have been identified as predictors of survival in advanced stages.^67^ Obese patients have 17% higher risk of dying from the disease than lean patients. Additionally, obese women have 30% higher risk of developing clear cell, mucinous, or endometroid ovarian cancers. Scarce evidence exists on the causal mechanisms, but it is surmised that excessive estrogen signaling is partially responsible.^20^ Our group demonstrated that obese women with HGSOC have poorer progression‐free and overall survival compared with the lean counterpart.^56^ We also demonstrated in two international cohorts (The Cancer Genome Atlas and Australian Ovarian Cancer Study) that HGSOC overexpressing obesity and lipid metabolism‐related genes have poorer oncologic outcomes.^57^ Obesity also impacts on secondary cytoreductive surgery and overall survival in women with recurrent disease.^68^ In relation to vulvar cancer, obesity was associated with a shorter time to recurrence in the AGO‐CaRE‐1 study and this was mainly attributed to a higher risk of local recurrence.^69^

## 7 NEGATIVE EFFECTS OF OBESITY ON OVERALL SURVIVAL OF GYNECOLOGIC CANCER SURVIVORS: RELEVANCE OF LIFESTYLE CHANGES

Perhaps the greatest health threat among gynecologic cancer survivors is weight gain over time or persistence of obesity after treatment completion.^70^ Studies demonstrated that women with endometrial cancer have significantly higher risk of mortality from other obesity‐driven diseases, such as heart disease or type 2 diabetes, compared with women without cancer.^70^, ^71^ A prospective report demonstrated that morbid obesity is associated with a significantly increased risk of death from several women's cancers. For women with a BMI of 40 or higher, the relative risk (RR) is 1.62 (95% CI, 1.40–1.87) and for BMI 35–39.9, the RR is 1.32 (95% CI, 1.20–1.44).^13^ Evidence suggests that weight management and physical activity improve overall health and well‐being and reduce the risk of morbidity and mortality among cancer survivors.^72^ Weight loss after bariatric surgery is more sustained than after other interventions and is protective against endometrial cancer.^6^

ASCO is highly committed to reducing the impact of obesity on cancer and the establishment of a multipronged initiative to accomplish this goal. Such an initiative considers: (1) increasing education and awareness of the evidence linking obesity and cancer; (2) providing tools and resources to help oncology providers address obesity with their patients; (3) building and fostering a robust research agenda to better understand the pathophysiology of energetic balance alterations, evaluate the impact of behavioral changes on cancer outcomes, and determine the best methods to aid cancer survivors in the implementation of effective and useful modifications to lifestyle and behavior; and (4) advocating for policy and systems change to address societal factors contributing to obesity and improve access to weight management services for cancer patients.^73^

## 8 ROLE OF OBESITY IN CONDITIONING RESPONSE OF FUTURE THERAPEUTIC VENUES IN GYNECOLOGIC CANCERS

The current therapeutic scenario is moving to the precision medicine. Beyond choosing the most effective surgery, radiotherapy, and/or chemotherapy scheme for any cancer, hopes are pinned on identifying cancer weaknesses, designing targeted therapies, and enhancing the host's antitumor immune response to improve clinical outcomes. However, the promising responses observed in preclinical models with some targeted therapies (i.e. immunotherapies) have not translated in the same results when challenged in gynecologic cancer patients.^74^ Factors contributing to impair their efficacy are aging, the composition of gut microbiome, and obesity.^75^ The additive effects of increased conversion of androgens into estradiol and estrone by peripheral hypertrophic adipocytes, increased bioactive estrogens, and increased insulin signaling in insulin‐resistant obese patients, which converge into the mitogenicPI3K/AKT/mTOR signaling pathway, are also to be taken into account. As such, addition of metformin and cholesterol‐lowering statins^76^ may aid in therapy response under specific circumstances. In addition, obesity and associated gut dysbiosis condition a tumor microenvironment where the antitumoral immune response becomes exhausted. Novel research on gene expression profiles and cell–cell communication networks is shedding new light on our understanding of the molecular and cellular wiring governing homeostasis and disturbed states in obesity. A recent study on single‐cell RNA sequencing and cell–cell ligand‐receptor interactome revealed that mature natural killer cells are depleted in the adipose tissue of obese compared with lean patients, and negatively correlated with patient BMI, with a relative increase of immature natural killer and tissue‐resident natural killer cells.^77^ These and other developing technologies, including lipidomics approaches,^21^ will continue to provide a detailed and unbiased cellular landscape of homeostatic and dysregulated circuits to further our understanding of health and disease, including obesity‐related disorders.

## AUTHOR CONTRIBUTIONS

IW and MC shared the concept design, literature review, and writing of the manuscript.

## CONFLICTS OF INTEREST

Relating to the submitted work, MC received a grant from Fondecyt nº 1201083. IW has no conflicts of interest to declare.
